# Alterations in Appetite-Regulating Hormones in Girls with Central Early or Precocious Puberty

**DOI:** 10.3390/nu15194306

**Published:** 2023-10-09

**Authors:** Galateia Stathori, Anastasia-Maria Tzounakou, George Mastorakos, Nikolaos F. Vlahos, Evangelia Charmandari, Georgios Valsamakis

**Affiliations:** 1 Center for the Prevention and Management of Overweight and Obesity, Division of Endocrinology, Metabolism and Diabetes, First Department of Pediatrics, Medical School, National and Kapodistrian University of Athens, ‘Aghia Sophia’ Children’s Hospital, 11527 Athens, Greece; g.stathor@gmail.com (G.S.); anastasia.tzounakou@gmail.com (A.-M.T.); 2 Second Department of Obstetrics and Gynecology, Medical School, National and Kapodistrian University of Athens, ‘Aretaieion’ University Hospital, 11528 Athens, Greece; mastorakg@gmail.com (G.M.); nfvlahos@gmail.com (N.F.V.); gedvalsamakis@yahoo.com (G.V.); 3 Division of Endocrinology and Metabolism, Center of Clinical, Experimental Surgery and Translational Research, Biomedical Research Foundation of the Academy of Athens, 11527 Athens, Greece

**Keywords:** central precocious puberty, children’s obesity, leptin, ghrelin, GLP-1, Nesfatin-1, Orexin-A

## Abstract

The prevalence of central precocious puberty (CPP) in girls has increased worldwide and is often associated with obesity in childhood as well as high fat/high glycemic index diets. Evidence suggests that subjects with obesity present with alterations in appetite-regulating hormones. The arcuate and paraventricular nuclei of the hypothalamus are the centers of action of appetite hormones, as well as the location of gonadotropin-releasing hormone (GnRH) neurons, the activation of which results in the onset of puberty. This anatomical proximity raises the question of possible alterations in appetite-regulating hormones in patients with CPP. Furthermore, diet-induced hypothalamic inflammation constitutes a probable mechanism of the pathophysiology of CPP, as well as alterations in appetite-regulating hormones in young children. In this article, we summarize the evidence investigating whether girls with CPP present with alterations in appetite-regulating hormones. We present evidence that leptin concentrations are elevated in girls with CPP, ghrelin concentrations are lower in girls with CPP, nesfatin-1 and orexin-A concentrations are elevated among girls with premature thelarche, and insulin concentrations are increased in girls with early menarche.

## 1. Introduction

Central precocious puberty (CPP) is characterized by the activation of the hypothalamic–pituitary–gonadal axis and the subsequent increase in concentrations of sex steroids before the age of 8 years in girls and 9 years in boys [1]. CPP may be a manifestation of syndromic conditions with the development of cerebral hamartomas and gliomas (e.g., Prader–Willi syndrome, Cowden syndrome) or central nervous system (CNS) abnormalities, such as infections, hydrocephalus, brain injury and cranial irradiation [2]. Puberty ensues following the activation of the gonadotropin-releasing hormone (GnRH) neurons, the majority of which are situated at the arcuate nucleus (ARC) and the medial preoptic area of the hypothalamus in humans [3]. 

Recent evidence suggests a tendency toward the earlier onset of puberty, particularly in girls [4,5,6,7]. At the same time, the prevalence of overweight and obesity in childhood and adolescence has increased dramatically during the last four decades. According to the World Health Organization (WHO), overweight and obesity are estimated to affect 41 million children younger than 5 years and more than 340 million children and adolescents aged 5–19 years [8,9]. The hypothalamus plays an important role in the pathophysiology of this multifactorial disease. Leptin, ghrelin, as well as other hormones that regulate food intake and energy expenditure, act on the hypothalamic ARC and the ventromedial hypothalamic nucleus (VMH) and regulate energy balance via strong reciprocal connections with the paraventricular hypothalamic nucleus (PVH) [10,11,12]. The small intestine-secreted hormone glucagon-like peptide-1 (GLP-1) regulates food intake via its action on hindbrain substrates. However, there is evidence that endogenous GLP-1 also acts on the PVH to suppress appetite and food intake [13,14]. Diet plays an important role in the development of obesity. More specifically, high-fat diets rich in calories account for the development of childhood obesity in most cases [15].

The prevalence of both childhood obesity and early CPP has been rising, and large retrospective studies have shown that CPP or early puberty is higher among girls with overweight or obesity. The mechanism of this association between obesity and CPP in girls has not yet been elucidated, while this positive correlation has also not been established in boys [16,17]. Interestingly, the consumption of a high-fat diet has also been positively correlated with CPP, independently of obesity [18,19].

Scientific evidence suggests that appetite-regulating hormones act on the reproductive system in various ways [20,21,22,23,24]. As mentioned above, most of the appetite-regulating hormones act on the ARC and the VMH of the hypothalamus to regulate food intake [10,11,12,13,14]. Interestingly, the ARC is the hypothalamic locus of the GnRH neurons, the early activation of which results in CPP [3]. This anatomical proximity raises the question of whether CPP coexists with disorders of appetite-related hormones. The aim of this review is to examine whether girls with early or precocious puberty present with disorders of appetite-regulating hormones owing to obesity, a high fat/high glycemic index diet or other unknown mechanisms.

## 2. Precocious Puberty and Appetite Molecular Disorders

The relationship between dietary patterns and CPP has been explored in several studies. A large Chinese population-based study, which included 15,937 children, documented information on food intake using a food frequency questionnaire (FFQ). Diet patterns were categorized into three types: “traditional diet”, “unhealthy diet”, and “protein diet”. A significant positive correlation between “unhealthy diet” and precocious puberty in boys and girls was observed [25]. Furthermore, Nguyen et al. showed that girls with an early onset of menarche had higher energy intake and higher consumption of polyunsaturated fatty acids [26]. Although environmental and socio-economic factors are associated with unhealthy diet patterns, higher intakes of energy are associated with impaired central hunger-satiety signaling too [27]. 

### 2.1. Leptin 

Since the discovery of leptin in 1994, many research studies have explored its role in the reproductive axis. Leptin deficiency, such as prolonged starvation, leads to decreased activation of the reproductive axis [28,29]. The mechanism of action of leptin in the reproductive axis is still debatable. Nevertheless, recent findings have proposed that its effects on the hypothalamus do not occur through direct interaction with GnRH neurons. Instead, evidence suggests that leptin’s actions are mediated indirectly through the activation of kisspeptin neurons, which subsequently stimulate the GnRH neurons [20,30]. 

Kisspeptin-neurokinin B- dynorphin (KNDy) neurons express neuropeptides that regulate GnRH release [31]. Scientific findings indicate that the KNDy neurons are influenced by the metabolic state. In a study conducted by True et al., it was demonstrated that rats experiencing a negative energy balance exhibited reduced levels of kisspeptin and neurokinin B in the ARC. The authors posited that this reduction could potentially contribute to decreased GnRH release and subsequently lead to reproductive dysfunction [32]. Harlow et al. showed that in female lambs, undernutrition reduced kisspeptin levels within the KNDy neurons, resulting in a decrease in LH and LH pulse amplitudes [33]. Yang et al. showed that both negative energy balance (fasting) and positive energy balance (diet-induced obesity) dysregulate the KNDy-associated gene expression in the ARC in female mice. The authors suggested leptin and ghrelin as probable communicators between energy homeostasis and KNDy-associated reproduction [34]. The latter is supported by multiple data confirming a relationship between leptin levels and kisspeptin expression: leptin-deficient mice have a lower Kiss1 mRNA expression in the ARC nucleus [35]. Also, in mice, 40% of the kisspeptin-expressing cells in the ARC nucleus of the hypothalamus express leptin receptors, suggesting that these kisspeptin neurons are leptin targets [36]. Qiu et al. showed that, in guinea pigs, leptin depolarizes kisspeptin neurons through the activation of transient receptor potential canonical (TRPC) channels [37]. The same team later proved that insulin acts on kisspeptin neurons in the same way. Interestingly, two animal studies have shown that recombinant leptin administration in female mice leads to early puberty [38,39].

Leptin concentrations tend to increase in normal girls entering puberty and decrease in normal boys entering puberty [40]. The cause of this difference is unknown, although it is most likely owing to the heterogeneity of body composition between the two sexes, which is induced by sex hormones. There is little information regarding leptin concentrations in patients with CPP. Palmert et al. determined the leptin concentrations in children with CPP and compared them with those of healthy subjects. They found that girls with CPP had elevated leptin concentrations than age-matched prepubertal children and that leptin concentrations were negatively associated with the body mass index (BMI) (mean leptin SD scores of girls with CPP: +0.5 ± 0.1 and +0.8 ± 0.2 compared to normal girls at Tanner stages 1–2 and 3–4, respectively, *p* < 0.002 for both). No difference in leptin concentrations was found between boys with CPP and prepubertal boys. The negative correlation between leptin concentrations and BMI in girls with CPP raised the hypothesis that a sufficient body mass leptin threshold is required for the initiation of puberty rather than elevated leptin concentrations. As for the non-elevated leptin concentrations found in boys with CPP, the authors suggested that the reproductive axis in females may be more dependent on fat indices than in males because of the child-bearing nature of females [41].

Su et al. compared leptin concentrations between 249 girls with CPP and 219 control girls. They found that leptin concentrations were significantly increased in the CPP group compared to the control group (7.6 ± 5.7 ng/mL vs. 6.2 ± 5.4 ng/mL, *p* < 0.01) and that, contrary to Palmert’s findings, leptin concentrations were positively correlated with BMI. They next studied whether higher concentrations of leptin in girls with CPP are caused by genetic variations of the leptin gene or the leptin receptor gene. For this purpose, DNA from both CPP and control groups was searched for 3 SNPs of leptin and the leptin’s receptor genes that have been associated with a higher bone mineral density and extreme obesity: the G > A variant in the leptin promoter at nt-2548 (rs7799039) and two SNPs in the leptin receptor Q223R (rs1137101) or K109R (rs1137100). The researchers concluded that there was no significant difference in the frequency of these SNPs between the CPP and the control group, suggesting that the higher leptin concentrations of the CPP group were probably not due to genetic causes [42]. 

Kang et al. compared 72 girls with a normal BMI with CPP (group A), 56 overweight and obese girls with CPP (group B) and 30 prepubertal girls with a normal BMI (NC, normal controls). Leptin concentrations were significantly higher in both group A and group B compared to the NC (group A = 4.21 ng/mL, group B = 5.64 ng/mL, NC = 2.35 ng/mL, *p* < 0.001), confirming that leptin concentrations are higher in girls with CPP independently of BMI. As expected, leptin concentrations were higher in group B than in group A. These findings further support the notion that sufficient leptin concentrations are important for the initiation of puberty [43].

Finally, another study compared leptin concentrations in 20 girls with CPP, 20 pre-pubertal girls with the same chronological age as the patients (group 1) and 20 girls with the same bone age, pubertal stage and BMI of the girls with CPP (group 2). Serum leptin concentrations in girls with CPP were similar to group 2 (9.0 ± 0.8 vs. 9.1 ± 0.9 ng/mL; ns) and higher than group 1 (5.6 ± 0.9 ng/mL, *p* < 0.001). In all three groups, leptin concentrations correlated significantly with BMI. These findings indicate that leptin concentrations in girls with CPP are higher than leptin concentrations in prepubertal girls of the same age and similar to those of older girls who have entered puberty normally [44].

All the above data suggest that leptin concentrations are increased in girls with CPP compared to age-matched prepubertal girls. Leptin concentrations in girls with CPP do not differ from those of older girls of the same pubertal stage. Studies have confirmed this correlation after BMI adjustment, suggesting that elevated leptin concentrations in CPP may not be associated with BMI. However, BMI is an inaccurate measure of body fat. Leptin concentrations are positively correlated to body fat mass in the general population. The BMI-matched girls included in these studies may have different fat/muscle distributions, accounting for the non-correlation of BMI and leptin. In any case, a sufficient threshold of leptin concentrations is probably required to initiate puberty rather than extremely elevated leptin concentrations, as indicated by the above studies. The pathophysiology and the mechanism of action of leptin in the initiation of CPP is not clear. It is well-known that during low food-intake periods (starvation, anorexia nervosa), the gonadotropic axis is suppressed, thereby protecting the individual from procreation and energy expenditure during limited food intake [45]. The restoration of leptin concentrations reactivates the gonadotropic axis. On the other hand, studies in mice showed that leptin administration caused early puberty [38,39]. There are two suggested hypotheses: 1. A relative excess of leptin causes the early activation of the gonadotropic axis and puberty 2. The excess of leptin is not the origin of CPP but co-exists with CPP due to a common underlying pathogenetic mechanism. Further studies are required to delineate the exact etiology. 

### 2.2. Insulin

Insulin, beyond its primary function as a blood glucose regulator, serves as a communicator of energy availability to the GnRH neurons of the hypothalamus. Mice with inactivated insulin receptors exhibit impaired spermatogenesis and ovarian follicle maturation due to the hypothalamic dysregulation of LH [46]. Hyperinsulinemia has been consistently associated with hyperandrogenemia and reproductive irregularities in women diagnosed with polycystic ovary syndrome (PCOS). However, the precise underlying pathophysiological mechanism has not been elucidated. [47]. Both in vivo and in vitro studies have demonstrated that insulin stimulates GnRH secretion, resulting in increased LH secretion. Interestingly, in the same studies, leptin was found to potentiate the action of insulin during GnRH secretion in vitro but had no significant effect when acting alone [48]. Although insulin receptors are present in the GnRH neurons in mice, a direct insulin-induced GnRH release modulation has not been confirmed [49]. Cernea et al. found that, in ewes, 94% of KNDy neurons bear insulin receptors and that prenatal testosterone administration results in a significant decrease in insulin receptors in these neurons. This finding suggests that insulin may exert its modulating control in the GnRH neurons via the KND neurons. Interestingly, no insulin receptors were found in ewes’ GnRH neurons, contrary to the findings in mice [50]. Another research team, while investigating insulin’s actions in the ARC of guinea pigs and mice, found that insulin directly stimulates the kisspeptin neurons via the activation of phosphatidylinositide3-kinase (PI3K) and the opening of transient receptor potential 5 (TRPC5) channels, similarly to leptin’s action [51]. This finding further supports the hypothesis that insulin indirectly regulates GnRH through its influence on KNDy neurons.

Few data are available on insulin plasma levels in girls with CPP. Sorensen et al. compared fasting insulin concentrations between girls with CPP, age-matched control girls and puberty-matched control girls. The CPP group had significantly higher fasting insulin concentrations, compared to both control groups [(Fasting insulin (pmol/L): CPP group = 58 (40–121), age-matched prepubertal control group = 33 (9–64), puberty-matched control group = 44 (11–101), *p* < 0.05)] [52]. Another longitudinal study showed that girls with early menarche also had higher insulin concentrations, as well as glucose intolerance compared to healthy girls, independent of BMI. Although it has been shown that, with the onset of puberty, insulin sensitivity decreases and insulin concentrations increase, the authors showed that girls with early menarche continued to maintain hyperinsulinemia and/or insulin resistance throughout puberty. These results could not be explained by BMI; however, in this study, girls with early menarche also had higher glucose concentrations, higher blood pressure and a higher percentage of body fat compared to the control girls [53].

### 2.3. GLP-1

GLP-1 is an intestine-producing hormone, mostly known for its capacity to stimulate insulin secretion in a glucose-dependent manner and to decrease food intake through its central hypothalamic actions. GLP-1 participates in the regulation of the reproductive axis. An in vitro study in immortalized LHRH-secreting cell lines and rat hypothalamic tissue showed that GLP-1 can stimulate the release of LHRH in both cases [54]. Furthermore, in male rats, (i) the intracerebroventricular injection of GLP-1 increased LH concentrations in vivo, and (ii) GLP-1 stimulated GnRH secretion in vitro. The same study showed that in male rats, GLP-1 concentrations in the hypothalamus decreased following a 48 h fast and returned to normal after refeeding. This finding indicates that in states of undernutrition, GLP-1 may mediate the disruption of the reproductive axis. However, the mechanism of action is still not clear [55].

Outeiriño-Iglesias et al. showed that GLP-1 administration in prepubertal rats induced vaginal opening and increased LH concentrations, resulting in the onset of puberty. Furthermore, the administration of GLP-1 in adult female rats resulted in a significant increase in both kisspeptin and kisspeptin receptor gene expression in the hypothalamus [56]. Similarly, Oride et al., through in vitro experiments using rat hypothalamic cells, showed that GLP-1 increases kisspeptin mRNA expression and that kisspeptin, in turn, stimulates the expression of GnRH, establishing kisspeptin as a key mediator in the regulation of the reproductive axis mediated by GLP-1 [57]. Interestingly, Heppner et al, using brain slices from mice, showed that during fasting conditions, GLP-1 administration fails to restore decreased LH levels, suggesting that the GLP-1 stimulatory effect on the reproductive axis is blunted in negative energy balance states [58]. As far as we are concerned, there are no data regarding GLP-1 concentrations in children with CPP. 

### 2.4. Cortisol

Elevated cortisol concentrations, as in long-term stress situations, can increase appetites and cravings for high-fat and sweet foods. An increased serum cortisol concentration is the result of the activation of corticotropin-releasing hormone (CRH) neurons in the PVN of the hypothalamus and the further release of ACTH from the pituitary gland. CRH neurons are mainly regulated by GABAergic signaling but receive signals from many neurotransmitter systems responding to stress [59]. Cortisol may act by stimulating the neuropeptide Y secretion while blunting leptin effects, creating a ‘leptin deficiency’ state and causing increased food intake [60,61]. A Chinese case–control study showed that cortisol concentrations during social stress were higher among girls with CPP compared to healthy girls. It should be noted, however, that in this study, the diagnosis of PP was based only on breast Tanner stages, without endocrinologic assessment to validate the activation of the GnRH axis [62]. 

### 2.5. Ghrelin 

Ghrelin is a hormone produced by enteroendocrine cells and is known to increase food intake through its central action on the hypothalamus. During food deprivation, ghrelin concentrations are increased. Studies in rats and monkeys have demonstrated that ghrelin participates in the regulation of the reproductive axis. The intracerebroventricular administration of ghrelin suppresses LH secretion in ovariectomized rats, while the peripheral administration of ghrelin decreases LH pulse frequency in ovariectomized monkeys [63,64].

Recently, Farkas et al. showed that GnRH neurons in mice bear growth hormone secretagogue receptors (GHS-R), also known as ghrelin receptors. The same team showed that ghrelin administration decreased the firing activity of GnRH in both male and female mice [65]. The intravenous administration of ghrelin in ovariectomized rats resulted in the down-regulation of kisspeptin expression in the medial preoptic area without affecting, however, kisspeptin expression in the ARC [66]. These findings propose that ghrelin, which is increased in food deprivation, plays a role in suppressing the reproductive axis in cases of undernutrition by reducing GnRH activity through its modulation of kisspeptin. 

The relationship between ghrelin concentrations and the premature activation of the gonadotropic axis has not received much attention. To date, there has only been one study that measured orexigenic hormone ghrelin concentrations in girls with CPP. Girls with CPP were compared to girls with premature thelarche (PT) and control girls. The CPP group had significantly lower ghrelin concentrations than the PT group and the control group [(CPP: Log (2.42 ± 0.26) ng/L, PT: Log (2.62 ± 0.21) ng/L, control: Log (2.58 ± 0.44) ng/L, *p* < 0.05)], which decreased with the progression of puberty and the advancement of Tanner stages [67]. 

As in the case of leptin, a decreased orexigenic hormone during the onset of puberty seems to offer no obvious natural benefit. Ghrelin acts on the ARC, which is the location of the GnRH neurons, the activation of which results in the onset of puberty. Thus, a common pathophysiological mechanism that affects the ARC could result in both CPP and alterations in ghrelin concentrations. 

### 2.6. Nesfatin-1

Nesfatin-1 potentially plays a role in the regulation of the reproductive system, but research findings in rats and fish present conflicting results. In female rats, the central administration of nesfatin-1 results in the significant elevation of gonadotropin concentrations [68]. The intraperitoneal injection of synthetic nesfatin-1 in goldfish resulted in an acute decrease in the expression of GnRH and the consequent inhibition of LH and FSH [69]. Furthermore, Rajeswari et al. recently showed that injection of a nesfatin-1-like peptide (a peptide exhibiting nesfatin-1-like metabolic effects) in fish resulted in the downregulation of the expression of kisspeptin and its receptor in the hypothalamus along with the downregulation of GnRH expression [70]. 

The relation between CPP and the anorexigenic neuropeptide nesfatin-1, as well as the orexigenic orexin-A has been studied recently. Almasi et al. compared nesfatin-1 serum concentrations between girls with PT and age-matched prepubertal girls. Both nesfatin-1 and orexin-A concentrations were significantly higher in girls with PT (without, however, PP) compared to the controls (*p* =  0.001). In this study, body weight, BMI, body fat mass, and basal metabolic rate (BMR) were all higher in the PT group compared to the control group [71]. Interestingly, in another study, nesfatin -1 and leptin concentrations were compared between non-obese girls with PT and age-matched prepubertal girls. Again, leptin and nesfatin-1 concentrations were significantly higher in the PT group compared to the control group [mean Leptin ± SD (pg/mL): PT group = 2.88 ± 0.74, control group = 2.34 ± 0.48, Mean Nesfatin-1 ± SD (ng/dL): PT group = 0.84 ± 0.61, control group = 0.44 ± 0.28, *p* < 0.05)], suggesting that these findings are independent of overweight or obesity. According to the authors, nesfatin-1 may control the reproductive axis centrally, depending on nutritional state [72]. 

## 3. Discussion

CPP has serious somatic and psychological repercussions. The early activation of the reproductive axis results in early epiphysial closure and a consequently shorter final height [73]. Early menarche is associated with an increased risk of breast cancer and cardiovascular disease [74]. Furthermore, early menarche has been associated with drug addiction, delinquency and the development of psychopathologies, such as depressive disorders, which persist through adult life [75]. Therefore, it is important to investigate the pathophysiology of CPP in order to prevent it. 

The correlation between CPP and obesity in girls is well-established. We also saw that there is a direct positive correlation between high-fat diets and CPP; however, evidence is still limited [25,26] (p. 2). Also, multiple studies suggest a causative role of the appetite-regulating hormones in the onset of CPP, as well as the probable co-existence of disorders of appetite-regulating hormones in children with CPP [41,42,43,71] (pp. 3–7). In this review, we examined whether girls with early or precocious puberty present with disorders of appetite-regulating hormones. 

The evidence suggests that leptin concentrations are elevated in girls with CPP compared to normal girls, while ghrelin concentrations are lower in girls with CPP, nesfatin-1 and orexin-A are elevated in girls with PT and insulin concentrations are higher in girls with early menarche. The cause of these alterations in appetite-related hormones in girls with CPP is not clear, alongside the pathophysiology of idiopathic CPP itself. However, there is evidence that appetite-regulating hormones are involved in the reproductive system by promoting the activation of the gonadotropic axis. Ahima et al. showed that leptin administration accelerates the onset of puberty in female mice [38] (p. 3). Sarah et al. proved that the intracerebroventricular injection of GLP-1 increased LH concentrations in male rats, and Outeiriño-Iglesias et al. proved that GLP-1 administration promoted the onset of puberty in rats by inducing vaginal opening and increasing LH concentrations [55,56] (p. 5). Furthermore, García-Galiano et al. showed that, in female rats, the central administration of nesfatin-1 results in the elevation of gonadotropin concentrations [68] (p. 6). Leptin acts on the reproductive axis via the hypothalamic kisspeptin system. It has also been proven that the administration of GLP-1 in female rats resulted in increased kisspeptin and kisspeptin receptor gene expression in the hypothalamus [56] (p. 5). The hypothalamic kisspeptin system appears to serve as the mediator for reproductive control regulated by other appetite-regulating hormones.

Interestingly, the correlation between alterations in appetite-regulating hormones and CPP has not been established in boys. Scientific interest, as indicated by the corresponding number of publications, has focused mostly on CPP in girls. This may be partly due to the fact that many studies use the clinical sign of thelarche as a marker of CPP, which is easily detectable. Another reason may be the fact that CPP in girls is 10 times more frequent than in boys worldwide [76]. Palmert et al. showed that in girls with CPP, leptin concentrations are elevated compared to healthy girls, whereas in boys with CPP, the correlation did not exist [41] (p. 3). Several studies have shown that leptin exerts its actions directly on the mammalian tissue and the ovaries: Leptin enhances the aromatase mRNA expression and its enzymatic activity in breast cells [77]. At the ovary level, leptin attenuates estradiol synthesis in human granulosa cells [78,79]. However, in vitro, leptin directly stimulates estrogen production by enhancing aromatase activity [80]. The literature concerning leptin action in the testes is much more limited; it seems that elevated leptin concentrations inhibit testicular steroidogenesis [81]. Thus, a probable resilience of males to developing alterations in appetite-regulating hormones and/or a deactivating role of leptin in the male reproductive system could also explain the non-confirmation of appetite hormone disorders in boys. In addition, scientific evidence proposes that the ghrelin-mediated suppression of GnRH neurons is dependent on estradiol. Conde et al showed that, in female mice, in states of elevated plasma ghrelin levels such as starvation, estradiol enhances the expression of ghrelin receptors in KNDy neurons, increasing their sensitivity to ghrelin and further suppressing the gonadotropic axis [82]. Yasrebi et al showed that, in both fed and fasted female mice, estradiol increases ghrelin receptor expression in the ARC. The same team showed that, in male mice, fasting increased ghrelin receptors in the ARC, whereas diet-induced obesity decreased ghrelin receptors in the ARC, suggesting that ghrelin signaling in the ARC behaves in a sex-dependent manner [83]. From the above findings, it occurs that ghrelin interacts with the gonadotropic axis in a distinct manner between males and females and that estradiol plays a role in mediating the effects of ghrelin on GnRH neurons. The apparent absence of alterations in appetite-regulating hormones in boys with CPP could potentially be explained by the divergent behavior of ghrelin between the two sexes. Ghrelin appears to function as a metabolic mediator of the reproductive axis, and this gender-specific interaction might account for the observed differences.

Studies in rodents have shown that a high-fat and/or high glycemic index diet causes a low-grade inflammatory response in the hypothalamus, indicating that the inflammation of the hypothalamus may be a contributing factor to the pathogenesis of obesity [84,85]. In 2012, P.Thaler et al. showed that obesity could be associated with increased gliosis of the mediobasal hypothalamus (MBH) [86]. In 2017, Kreutzer et al. demonstrated that patients with obesity exhibited T2 hyperintensity in the left MBH (indicating inflammation and gliosis), which was strongly associated with systemic low-grade inflammation (positive correlation of systolic IL-6 and CRP) [87]. The MBH includes the ARC nucleus, which is the center of action for most hormones that regulate appetite. The majority of GnRH neurons are also situated in the ARC nucleus. This anatomical proximity, along with the positive correlation of high-fat diets/obesity with CPP or early puberty, indicates that the diet-induced inflammation of the ARC could be a possible pathogenetic mechanism that is responsible for both the appearance of CPP and alterations in appetite-regulating hormones. However, diet-induced hypothalamic inflammation has been confirmed in both sexes; thus, this hypothesis does not explain why alterations in appetite-regulating hormones have not been equally confirmed in boys as in girls. 

The few existing human studies suggest that girls with CPP present with alterations in appetite-regulating hormones. However, there are a few limitations. Firstly, there is an obvious heterogeneity among the available surveys regarding clinical characteristics studied. For example, many research teams studied appetite hormones in girls with PT and not CPP. However, PT is not always consistent with CPP, and the true activation of the gonadotropic axis is not guaranteed. Secondly, among the available studies, it is unclear whether the alterations in appetite-regulating hormones pre-existed or appeared after the onset of puberty. As the order of appearance of appetite-regulating hormone disorders and CPP is unknown, it remains unclear whether these two phenomena derive from a common pathological process or if appetite hormone disorders independently trigger the onset of CPP. Finally, the possibility of alterations in appetite-regulating hormones in boys with CPP has not been established; therefore, we cannot conclude whether the phenomenon affects both sexes equally or just females.

In summary, the present review discussed data that imply that girls with CPP present with alterations in appetite-regulating hormones. The mechanism of this association is not known yet. As obesity is common among girls with CPP, the first hypothesis is that obesity leads to CPP and alterations in appetite-regulating hormones via common metabolic pathways. However, research has also shown the existence of alterations in appetite-regulating hormones in non-obese girls with CPP. Thus, the second hypothesis is that a separate mechanism, unknown so far, affects both the GnRH neurons promoting CPP and the hypothalamic appetite centers, leading to alterations in appetite-regulating hormones. Further research studies are needed to a gain better understanding of the pathophysiology of appetite-related disorders in girls with CPP in order to develop new therapeutic approaches for this important, constantly increasing medical condition. The existence of the same relationship in boys with CPP should also be explored. To our knowledge, this information is not currently available. It also needs to be established whether appetite-regulating hormone alterations in patients with CPP are reversible after diet adjustment or weight loss is not clear. To verify this, diet-modified interventional studies must be performed to explore first whether CPP reverses with a low-fat diet and second whether alterations in appetite-regulating hormones normalize.
